# Acquired Syphilis

**Published:** 1907-11

**Authors:** W. E. Hughes

**Affiliations:** Pocahontas, Ark.


### ACQUIRED SYPHILIS.

By W. E. HUGHES, Pocahontas, Ark.

Syphilis has been known to the human family since the birth of Christ and destroys more lives, homes and happiness; brings more suffering, poverty and disgrace into the circles of homes than any other disease known to the medical profession.

Syphilis is a constitutional disease, due to inoculation with specific virus; the first symptom which is the chancre, which makes its appearance from two to five weeks after exposure.

The chancre or primary sore is commonly found about the corona glandis, but may appear any where on the body, contracted directly, by contact with chancre or secondaries, or indirectly from articles used by syphilitics.

It appears as an indurated pappule, which develops into an abrasion, tubercle or ulcer, the characteristics of which are, indurated base and thin, scanty secretions, inflammation slight around the sore, usually single not autoinoculable.

Buboes are polyganglinonic and painless; rarely suppurate and appear after an incubation period and is followed by secondaries. The Hunterian chancre is characterized by a greater depth, freer discharge and more marked induration.

The second stage consists in general enlargements of the glandular system, eruptions of the skin, which may assume different degrees of severity and appearance, and at times inflammation of the iris or periosteum, falling of the hair and a thousand and one other symptoms, too numerous to mention in this paper.

The pathology of secondary syphilis consists of congestion, infiltration and ulceration.

The development of these secondaries is preceded by malaise, fever and anemia, lasting for a few days and disappearing on the appearance of the eruption and sore throat.

The eruption of the skin may assume the appearance of crythema, lichen, vescular, bulous or pustular lesions; so in diagnosing any of the above skin diseases it is a good idea to be on the lookout for syphilis.

In the mucous membrane we have congestion, as in the sore throat, then infiltration with maceration of epithelium (mucous patches) and finally ulcers.

Some of the most striking features of the skin eruptions are, absence of itching, symmetrical arrangement on the body, reddish brown color polymorphous. Many kinds of eruptions on the body at the same time and on fading turns almost white.

The mucous patches consists of a congested, infiltrated maccule, the surface of which is from its peculiar position, continually moist, consequently becomes sodden and appears as an elevated flat maccule covered with a dirty offensive exudation.

Tertiary syphilis consists of the gumma which has no tendency to spontaneous cure and is characterized by the formation of masses of cells which infiltrate the surrounding tissue and breaks down in the centre.

A gumma may break down leaving an ulcer, or may be absorbed leaving a fibroid thickening or scar.

The gumma may attack the periosteum, causing nodes, caries or necrosis. If it involves the cutaneous and mucous surface, ulcers are found.

These ulcers of tertiary syphilis are symmetrical, and are not contagious. The tertiary ulcer begins as a gumma or lump, which when it breaks exposes a gray slough surrounded by granular tissue and the edges are rounded and sharply cut and have no tendency to heal except after receiving a specific treatment.

It is a fact that syphilis attacks every part of the body, bones, nerves, muscles, blood vessels and in fact, all parts of man, and is transmitted from parents to their offspring; however, I will not deal with any of the complications, but outline briefly my usual form of treatment.

We should never under any circumstances, begin treatment in a supposed case of syphilis until we have thoroughly substantiated our diagnoses, then my usual form of treatment is as follows:—

In the primary stage have the patient wash several times daily with black wash and dust the affected parts with calomel, iodel, or iodiform or some similar preparation.

In the second stage the usual treatment is with mercury, and I give the protoiodide on account of its convenience, varying the dosage according to the age and vitality of my patient and severity of the disease, for fifteen or twenty months, with any additional treatment I deem necessary.

After the secondaries subside I give iodide potash in a solution of chloride and bichloride of mercury for two or three years until every vestige of disease has vanished.

In conclusion, as a prophylaxis I believe the medical profession should urge congress to enact a law compelling every man infected with syphilis to be castrated; and every woman to have an ovariotomy for the future benefit of the human family; also I would indorse a law prohibiting any syphilitics landing on the soil of our grand old republic.

